# Growth performances, carcass traits, meat quality, and blood metabolic parameters in rabbits of local Algerian population and synthetic line

**DOI:** 10.14202/vetworld.2019.55-62

**Published:** 2019-01-08

**Authors:** Rafik Belabbas, María de la Luz García, Hacina Ainbaziz, Nadia Benali, Ali Berbar, Zoubeida Boumahdi, María José Argente

**Affiliations:** 1 Biotechnology Laboratory of Animal Reproduction, Institute of Veterinary Sciences, Blida; 2 Departamento de Tecnología Agroalimentaria, Universidad Miguel Hernández de Elche, Alicante, Spain; 3 Laboratory of Research Health and Animal Production, National Veterinary School, Algeria

**Keywords:** carcass traits, growth, metabolic profile, rabbit, synthetic line

## Abstract

**Aim:**

The objective of this work was to study the growth performance, slaughter traits, meat quality, and metabolic profile in rabbits of local Algerian population and a synthetic line.

**Materials and Methods:**

In total, 120 weaned rabbits were used (60 per group). Growth traits were recorded from weaning (35 days) to slaughter (91 days). At slaughter, carcass traits, meat quality, and metabolic profiles were measured.

**Results:**

The synthetic line showed heavier total weight and faster daily weight gain than the local population (+15% and +19%, respectively), better feed conversion (3.92 vs. 4.81 g/g), and heavier weight of cold carcass, and perirenal fat (+15%). No differences were found between the two groups in dressing out percentage, muscular pH, weight of liver, or scapular fat. Wider intestinal villi were found in the synthetic line (+20%, p<0.0001) allowing better absorption surface in this line. The synthetic line also showed higher fat content (3.41% vs . 2.22%, p<0.0001) in the meat and lower protein content (22.02% vs . 18.98%, p=0.0002). Glucose level was 19% higher in the local population than in the synthetic line.

**Conclusion:**

The synthetic line is well adapted to the local conditions of Algeria. This line has shown better growth, daily gain, and feed conversion, due to its better intestinal absorption surface.

## Introduction

Most countries have one or more local rabbit breeds that could play an important role in commercial production [1]. In Algeria, traditionally, rabbit meat production has been conducted with local populations [2]. Since 1990, the Technical Institute for Animal Production (ITELV) and Tizi Ouzou University have set up programs for the characterization of these populations [3,4]. These studies highlighted their good ability of adaptation to local conditions, particularly their suitability for production in hot stress conditions. However, the weight of these adult rabbits and their litter size are too low to allow them to be considered for intensive production. The creation of synthetic lines has been adopted as a new strategy to improve rabbit production in hot climate countries, such as in Egypt and Saudi Arabia [5,6]. These lines were developed by cross-breeding between foreign commercial lines and local breeds, with the aim of reaching a compromise between the performance of the foreign commercial lines and the adaptation to the heat of the local population [7].

To develop rabbit meat production in Algeria, a synthetic rabbit line was created since 2003 by cross-breeding females from the local population with males from the French INRA 2666 strain at ITELV [8]. Recently, Zerrouki *et al*. [9] and Sid *et al*. [10] compared the synthetic line to the local Algerian population. This new synthetic line has shown a 20% more litter size than the local Algerian population, which is mainly due to their improve ovulation rate [11]. Several studies have been performed to assess the reproduction performance of the synthetic line [10-12]. However, there are no studies about its growth performance, carcass traits, or meat quality.

The objective of the present work was to study the growth performance, carcass characteristics, intestinal morphometry, and some biochemical blood parameters in rabbits from the synthetic line and the local Algerian population.

## Materials and Methods

### Ethical approval

This study was approved by the Scientific Council of Biotechnology Laboratory of Animal Reproduction, University of Saad Dahlab Blida, Institute of Veterinary Sciences (Algeria).

### Animals, housing, and diet of growing rabbits

The experiment was carried out at the rabbit unit of the Saad Dahlab of Blida University, Algeria. A total of 120 rabbits of both sexes were used in this experiment, 60 animals from the local Algerian population and 60 from the synthetic line. All animals came from the homogenous litters (7-8 kits at weaning). The local Algerian population was generated from breeding stock received from different Algerian regions in 1988 [13]. The synthetic line was created by cross-breeding rabbits from the local Algerian population and the INRA 2666 strain in 2003 [8]. The rabbits used in this experiment belong to the 7^th^ generation of selection on litter size and body weight at 77 days.

The rabbits were weighted at weaning (35 days) and placed in individual wire mesh cages (44 cm×24 cm×31 cm) arranged in a flat deck layout. During the 8 weeks of the experiment, the rabbits were fed a commercial pelleted diet *ad* *libitum*. The composition and the chemical analysis of the diet are presented in Table-1 [14]. The chemical analysis of the diet was performed using recommendations of AFNOR [15]. Water was available *ad* *libitum* using nipple drinkers.

**Table-1:** Composition and chemical analysis of the diet.

Ingredient	Percentage
Corn grain	10.0
Barley grain	22.0
Wheat bran	14.5
Soybean meal	12.5
Dehydrated alfalfa	39.0
Limestone	0.4
Dicalcium phosphate	0.3
Sodium chloride	0.3
Premix*	1

**Chemical composition (% DM)**	**Percentage**

DM	90.2
Crude protein	16.1
Crude fiber	15.1
Ether extract	2.6
Ash	8.3
Neutral detergent fiber	34.2
Acid detergent fiber	17.3
Acid detergent lignin	3.9
Digestible energy (Kcal/kg)^#^	2562

DM=Dry matter,

* Mineral and vitamin composition (g/kg premix)=Se - 0.025, Mg - 5, Mn - 7.5, Zn - 7.5, I - 0.12, Fe - 3.6, Cu - 2.25, Co - 0.04, thiamin - 0.1, riboflavin - 0.45, calcium d-pantothenate - 0.6, pyridoxine - 0.15, biotin - 0.0015, nicotinic acid - 2, choline chloride - 35, folic acid - 0.4, Vitamin K_3_-0.2, dl-α-tocopheryl acetate - 1.35, cyanocobalamin - 0.0006, Vitamin A - 850000 IU, Vitamin D3-170000 IU,

# Digestible energy was estimated using the equation cited by Fekete and Gippert [14].

### Growth performance traits

To calculate the feed conversion ratio and average daily gain, food consumption, and live weight were measured weekly during the fattening period. Weighing was carried out before the morning feeding (at 8:00 h). The mortality was recorded daily. Data from dead animals were not included in the analysis.

### Slaughter and carcass composition traits

At the end of the experiment period (91 days), slaughter yield and carcass quality measurements were performed on a total of 35 animals for each group without prior fasting. The animals were chosen randomly since there is no sexual dimorphism at this age [16].

The slaughtering and carcass dissection procedures followed the World Rabbit Science Association recommendations described by Blasco *et al*. [17]. Immediately before slaughtering, the rabbits were weighted (slaughter weight, SW). The slaughtered rabbits were bled, skinned, and emptied of the digestive tract and urogenital organs before being weighted. The tail, fore, and hind legs were kept to conform to the regulations of commercial carcass presentation in Algerian markets [18]. The carcasses, with the head, thoracic cage organs (heart, lungs, thymus, trachea, and esophagus), liver, kidneys, and the perirenal and scapular fat, were weighed 30 min after slaughter and subsequently chilled at 4°C for 24 h. After this time, the weight of the chilled carcasses (CC) was measured, and the dressing out percentage was calculated (CC as a percentage of SW).

For the next set of measurements, the head was separated from the carcass and the liver, kidneys, and the perirenal and scapular fat were removed. The carcass was the cut between the last thoracic and the first lumbar vertebrae and between the 6^th^ and 7^th^ lumbar vertebrae, resulting in three parts: Fore, intermediate, and hind. The proportions to CC were calculated for the skin, full gastrointestinal tract, head, fore, intermediate, and hind parts. To determine meat to bone ratio, the left hind legs were dissected to separate bone from meat, and the weight of these parts was recorded [19].

### Intestinal morphometry

From the middle part of the jejunum, a 4 cm sample was excised (n=20 per group). The samples were placed in a 10% buffered neutral formaldehyde solution (pH 7.2) and routinely processed and embedded in paraffin wax for histology; sections are stained by the hematoxylin and eosin procedure [20]. Villus height, width, and the ratio height/width were measured on 30 vertically oriented villi per animal, using a light microscope connected to computer-assisted image analysis (Motic image plus 2.0).

### Meat quality measurements

Muscular pH of *Longissimus* *dorsi* and *Biceps* *femoris* was measured at the 5^th^ lumbar vertebra in the CC (n=35 per group) using a portable pH meter equipped with a glass electrode (3 mm diameter conic tip) suitable for meat penetration. The chemical composition of meat in the right hind leg was determined (n=15 per group) [21]. Dry matter (using an air-evacuated oven for 16 h), crude proteins (N×6.25), ether extract, and ash were determined according to the AOAC [22].

### Blood measurements

A blood sample was collected from each animal during slaughtering in heparinized tubes and then centrifuged at 3000 rpm for 10 min (n=35 per group). The plasma was collected and stored at −20°C until analysis. Plasma glucose, cholesterol, triglycerides, total proteins, creatinine, and urea were measured using a spectrophotometer (LKB Novastec) and available commercial kits (SPINREACT, SA, Spain) according to the manufacturers’ instructions.

### Statistical analysis

Growth performance, slaughter and carcass traits, meat quality, and plasma biochemical parameters were analyzed using the General Linear Models procedure of the SAS statistical package (v9.4, SAS Institute Inc., Cary, NC, USA 2017). Data were analyzed as a completely randomized design with genotype as the only source of variation.

## Results

### Health status and growth performance

Survival rate during the fattening period was similarly high (around 95%) in both groups (p (χ^2^)=0.61), especially taking into account that neither antibiotics nor probiotics were used in the whole experiment. According to the autopsies, the main cause of mortality was a digestive disorder with diarrhea as an external symptom.

Weight at weaning was not affected by the genotype of the animal (Table-2). The synthetic line showed a higher live weight at 91 days than the local population (+15%, p<0.0001). No significant difference was found for the average daily feed intake between genotypes. Average daily gain and feed conversion ratio were 19% and 18% higher in the synthetic line than in the local population, respectively (p<0.0001).

**Table-2:** Growth performance from 35 days of age until slaughter in the local population and the synthetic line.

Traits	Local population (n=57)	Synthetic line (n=55)	SEM	p-value
Live weight at 35 days, g	566.05	552.80	59.82	0.243
Live weight at slaughter (91 days), g	2101.86	2462.05	165.82	<0.0001
Average daily feed intake, g	132.05	134.09	16.74	0.521
Average daily gain, g	27.49	34.09	2.47	<0.0001
Feed conversion ratio	4.81	3.92	0.43	<0.0001

SEM=Standard error of the mean

### Carcass traits

Carcass traits are summarized in Table-3. The synthetic line had a lower maturity rate at slaughter than the local population (−3%, p=0.002) and had heavier skin (+23%, p<0.0001). The two lines had a similar weight of full gastrointestinal tract. The hot and cold carcasses from the synthetic line were heavier than those from the local population (+15%, p<0.0001). No differences were found in the dressing out percentage, liver, and scapular fat weights. However, the synthetic line exhibited higher perirenal fat weight than the local population (25 g vs . 21 g p<0.001). The height of intestinal villus was similar in both genotypes, but its width was higher in the synthetic line (+20%, p<0.0001). Therefore, ratio of height: width of villus was smaller in the synthetic line than in the local population (7.3 vs. 5.8, p<0.0001). The genotype did not influence pH measured in the *L*. *dorsi* or *B. femoris*. The fore, intermediate, and hind parts of the carcasses were heavier in rabbits from the synthetic line compared to those from the local population (+20%, +15%, and +14%, respectively; p<0.0001). Ratio of liver and scapular fat to cold carcass was higher (p<0.0001) in the synthetic line (4.88% vs. 4.28% and 0.54% vs. 0.43%, respectively). Moreover, the synthetic line exhibited a higher ratio of fore part to cold carcass than the local population (+8%, p<0.0001). No differences were observed in ratios of perirenal fat depots, intermediate parts, and hind parts to cold carcass. Meat-to-bone ratio was also similar in both lines.

**Table-3:** Slaughter data, carcass traits, and jejunum morphometry in the local population and the synthetic line.

Traits	Local population (n=35)	Synthetic line (n=35)	SEM	p-value
Maturity rate, %	72.88	70.41	3.20	0.002
SW, g	2113.74	2464.42	99.04	<0.0001
Skin weight, g	199.37	257.71	48.26	<0.0001
Full digestive tract, g	305.91	332.91	72.29	0.123
HC weight, g	1454.54	1716.34	59.24	<0.0001
CC, g	1415.08	1674.08	60.40	<0.0001
Dressing out percentage (CC/SW), %	67.10	68.05	4.21	0.349
Liver, g	69.18	71.65	8.97	0.253
Scapular fat, g	7.75	7.35	1.52	0.276
Perirenal fat, g	21.25	25.12	3.37	<0.0001
Jejunal morphometry				
Villus height, μm	1087.48	1070.05	139.35	0.603
Villus width, μm	149.11	186.80	20.50	<0.0001
Ratio Height/Width	7.43	5.76	1.18	<0.0001
Muscular pH				
*Longissimus dorsi*	5.61	5.63	0.059	0.181
*Biceps femoris*	5.80	5.76	0.11	0.142
Carcass division				
Fore part, g	447.92	575.44	41.53	<0.0001
Intermediate part, g	243.28	286.76	41.88	<0.0001
Hind part, g	467.59	541.50	40.83	<0.0001
Proportions				
Skin, % SW	9.39	10.40	1.74	0.018
Liver, % CC	4.88	4.28	0.54	<0.0001
Scapular fat, % CC	0.54	0.43	0.10	<0.0001
Perirenal fat, % CC	1.50	1.50	0.23	0.982
Fore part, % CC	31.63	34.36	2.19	<0.0001
Intermediate part, % CC	17.186	17.09	2.42	0.871
Hind part, %CC	33.07	32.39	2.84	0.318
Meat-to-bone ratio	7.25	7.29	0.62	0.757

SW=Slaughter weight, HC Hot carcass, CC=Chilled carcass, SEM=Standard error of the mean

### Chemical meat composition

Table-4 shows meat composition at 91 days in the synthetic line and the local population. The genotype did not affect the ash content in the meat. Moisture and fat contents were higher in the synthetic line than in the local population (+9% and +35%, respectively, p<0.0001). However, crude protein was lower in the synthetic line (22% vs . 19%, p=0.0002).

**Table-4:** Chemical meat composition in the local population and the synthetic line.

Traits	Local population (n=15)	Synthetic line (n=15)	SEM	p-value
Moisture, %	62.33	68.81	3.51	<0.0001
Dry mater, %	38.07	31.18	3.18	<0.0001
Crude protein, %	22.02	18.98	1.91	0.0002
Ether extract, %	2.22	3.41	0.59	<0.0001
Ash, %	1.86	1.92	0.30	0.6007

SEM=Standard error of the mean

### Plasma metabolic parameters

Plasma biochemical parameters are shown in Table-5. The synthetic line had lower glycemia than the local population (5.92 mmol/L vs . 7.32 mmol/L, p<0.0001). However, the plasma concentrations of cholesterol, triglycerides, total proteins, creatinine, and urea were similar in both groups.

**Table-5:** Plasma values of biochemical parameters in local population and synthetic line.

Traits	Local population (n=35)	Synthetic line (n=35)	SEM	p-value
Glucose, mmol/L	7.32	5.92	1.38	<0.0001
Cholesterol, mmol/L	1.80	1.77	0.30	0.744
Triglycerides, mmol/L	1.70	1.77	0.26	0.300
Total proteins, g/L	62.38	63.52	7.94	0.547
Creatinine, μmol/L	93.36	92.22	10.97	0.665
Urea, mmol/L	12.46	12.72	1.41	0.450

SEM=Standard error of the mean

## Discussion

### Health status and growth in the fattening period

The health status of the animals can be considered good despite the experiment being conducted without using antibiotics. The mortality rate in both genotypes was similar to that reported in previous studies by Lakabi [23] and Benali *et al*. [24] but lower than that reported by Gacem *et al*. [25]. Lower mortality in our populations would be related to more stringent hygiene conditions applied in the rabbity, as well as to a better food quality [23]. The majority of losses were recorded during the 1^st^ week after weaning. The dead animals presented diarrhea as an external symptom, probably due to digestive disorders related to the stress caused by the weaning process (i.e., separation from the mother and the change of food). Similar observations have been reported by Knudsen *et al*. [26]. Food restriction has been proposed as an effective method for reducing post-weaning digestive disorders [24], as well as providing diets with highly-digestible fiber around weaning [19].

Weight at weaning was similar between the synthetic line and the local population, and it is within the range reported by Gacem *et al*. [25] and Sid *et al*. [10]. At 13 weeks of age, live weight in the local population was 14% higher than that reported by Lounaouci [18] and Benali *et al*. [24]. This improvement could be related, on the one hand, to the use of individual cages which led to the elimination of competition between animals [27], and on the other hand, to the excellent quality of food used in this study compared to the one used by these authors, which was lower in fiber and protein. The synthetic line was 15% heavier than the local population. The results of this study show the interest of selection of this line.

Average daily feed intake was unaffected by the genotype, and it was similar to that reported by several authors on different local populations and strains [15,28]. Our local population showed a similar average daily gain than other local populations [24,29]. However, the average daily gain was 19% higher in the synthetic line, but it was still lower than gains of different reported in other lines such as Hyla line (−16%) [30], New Zealand rabbits (−6%) [31], and V line (−11%) [28]. On the other side, although daily feed intake was similar in both groups, feed ratio conversion was better in the synthetic line (3.92) than the local population (4.81) due to a better feed efficiency in this line [23].

### Dressing and carcass characteristics

Maturity degree was estimated considering the adult weight of 2900 g and 3500 g for the local population and the synthetic line, respectively [18,25]. In both groups, maturity degree was higher than those reported in others lines by 55-60%. This could be related to the slaughter at a younger age (77 days vs. 91 days) [31]. Our findings indicated that it is possible to slaughter animals before 13 weeks of age, but the carcasses are too light to be marketed.

Skin weight and its percentage to live weight at slaughter were in the same range of those obtained in other populations [23,24]. In this study, they were higher in the synthetic line compared to the local population. The lightweight of the skin in the local population was hypothesized as characteristic of this population by Berchiche *et al*. [32], possibly due to an adaptation to the thermal stress conditions in which the local population has been brought up traditionally.

Dressing out percentage is a very important economic trait in the rabbit market [33]. The drop out percentage in our experimental groups was similar and higher than the standard values for medium-size rabbits [34]. It appears that the difference in degrees of maturity between groups was not large enough to cause significant changes in dressing out percentage, which usually increases with the degree of maturity [34]. In contrast, it was higher than those reported by several authors on different rabbit strains [35], which could be related to the late slaughter (13 weeks) decreasing the proportion of the digestive tract and consequently improving the drop out percentage [36]. The variation in dressing percentage among studies might be related to the use of different genotypes [37], feed quality [38], and the live body weight at slaughter [39]. In addition, the carcass definition varies from country to country. For instance, in Europe, the head and feet are part of the carcass, so the rabbit dressing percentage obtained (60-62%) is higher than that in the United States (50%) where head and feet are removed.

There was no difference for liver weights between groups, but a higher percentage of liver was found in the synthetic line compared to the local population. The variation in liver percentage among genotypes was already reported by several authors [21,33]. It appears to be due to the difference in food ingestion between animals [40] rather than the difference in maturity degree. In fact, the growth of liver is almost isometric [41]; thus, similar percentages should have been found regardless of the degree of maturity of the animals. However, in this study, the animals had similar food ingestion.

The local population and the synthetic line have shown lower adiposity compared to the optimum (3%) in the standard commercial lines [42]. There was no difference in scapular fat weight between groups. However, the percentage of scapular fat to cold carcass was higher in the local population. On the other side, the synthetic line rabbits have shown heaviest perirenal fat weight (+15%). These results could be a consequence of higher live weight of the synthetic line than the local population as observed by Deltoro and Lopez [43].

Moreover, according to Larzul *et al*. [44], the selection can modify the fat depots. Indeed, rabbits selected for growth have in general higher adiposity. The increase in fat tissue as a consequence of selection for growth rate has been also seen in poultry [45]. It should be noted that rabbit carcasses have a small dissectible fat content [46] and this is not normally used as measure for quality. Therefore, other criteria are used to define rabbit carcass quality such as the meat percentage in the carcass and muscularity defined as the meat-to-bone ratio.

In the present experiment, the jejunal villi height and width were close to those observed in the study by Benali *et al*. [24] in rabbits of the same age (91 days). However, Romero *et al*. [47] observed shorter villi at the age of 63 days. Similar findings have been observed by Gallois [48] who confirmed the change in the intestine villi measurements within the age of the animal. Furthermore, well-developed villi are an indicator of good health status at the farm because Dewrée *et al*. [49] observed shorter villi in ill rabbits. Villi of rabbits from the synthetic line were wider than those from the local population (+20%) allowing better surface absorption in the former line. In fact, Wiese *et al*. [50] reported that the height of the villus is not indicative of the total size of the absorption surface, which depends on the shape of the villi. A shorter and wider villus can present a larger surface absorption than higher but narrower villus.

The pH value represents a key role in the preservation of meat quality during storage. In fact, it determines environmental microbial balance. In this study, the pH was in the range between 5.61 and 5.80, so the meat from these rabbits could be considered suitable for storage because pH value above 6 can stimulate the development of proteolytic microorganisms Zotte [51]. The pH of the meat has an important influence on other meat quality parameters such as holding water capacity, protein fractions, color, and tenderness [52]. In the present investigation, the mean values of *L*. *dorsi* or *B. femoris* pH were comparable to those obtained by several authors in rabbits [21,33]. In addition, pH was slightly higher in the *B. femoris* than in the *L*. *dorsi* because the former is considered more oxidizable muscle group [33]. We did not find a significant difference in pH for any of the muscles between the synthetic line and the local population. These results are in disagreement with the findings of Pla *et* *al*. [33] pointed out a variation in muscular pH within the genetic origin. However, Hernández *et al*. [53] did not find a difference in the muscular pH between lines when comparing animals at the same age.

The synthetic line has shown the higher weight of retail cuts than the local population thanks to its higher weight at slaughter. Similarly, the percentage of the fore part to cold carcass was higher in the synthetic line than in the local population, but the percentages of intermediate and hind parts to the cold carcass were similar in both groups. All these percentages were similar to those reported in the local population [24] and different INRA strains [4]. The weight of different retail cuts of the carcass change as the degree of maturity increases because they have different allometric coefficients [43]. However, in our case, increased maturity only affects the percentage of fore part.

Meat-to-bone ratio of the hind leg is the best predictor for the meat-to-bone ratio of the carcass [21]. Under our conditions, the meat-to-bone ratio was similar in both experimental groups and close to those reported by Lounaouci [18] in our local population (6.7) and in the synthetic lines used in European farms (5.3-6.5) [34]. The difference in meat-to-bone ratio can be related to the difference in the degree of maturity between animals [54].

### Meat quality

In relation to meat chemical composition, the synthetic line contained more moisture than the local population. Other authors have found a large variation in meat water content among breeds [54]. A lower degree of maturity and higher SW of the synthetic line might be associated with higher moisture content, lower protein content (−14%) and higher lipid content (+35%) than the local population, as highlighted in others strains by Zotte *et al*. [55].

### Blood metabolic profile

Glycaemia was higher in local rabbit (+19%). It is known, that glucose is increased in heat stress condition, and this increase can decrease cells’ ability to metabolize carbohydrates, accompanied by progressive proteolysis as an alternative process for energy production [56]. Furthermore, elevated plasma glucose levels in rabbits are generally due to various stress factors, such as stress blood collection [57]. Furthermore, blood cholesterol remains stable in heat stress condition, suggesting that liver tissue is not damaged [56]. Both lines show similar blood cholesterol level, triglycerides, and liver weight. Hence, the synthetic line is well-adapted to produce in heat condition.

## Conclusion

In summary, the synthetic line presented higher weight and daily gain as well as lower feed conversion ratio. The main difference in the carcass traits was found in the weights of skin, perirenal fat, carcass, and its different retail cuts. However, the dressing out percentage was similar in both groups. Rabbits from the local population have shown the best meat quality (low in fat and rich in protein). A similar metabolic profile between the local population and the synthetic line was found showing the good adaptation of this line to produce in a hot climate country such as Algeria.

## Authors’ Contributions

RB, HA, ZB, and MJA designed all steps of the study. MJA analyzed the data, MG and NB reviewed the manuscript, and RB wrote the manuscript draft and collected all data with AB. All authors read and approved the final manuscript.
